# Supplementary material for: A methodology for the design of experiments in computational intelligence with multiple regression models
Source: PeerJ. 2016 Dec 1;4:e2721. doi: 10.7717/peerj.2721 (PMC5136129; doi:10.7717/peerj.2721)
Supplement: Data S1 — Datailed results from UC Irvine Machine Learning Repository (Housing, Machine CPU, Wine Quality, Automobile and Parkinson) and the 3 Use Cases (Protein Corona, Gajewicz Metal Oxides and Aquatic Toxicity) [file peerj-04-2721-s001.zip › 6_UseCases/2_GajewiczMetalOxides/RFRFE.details.csv.repeatedcv.split2.pdf]

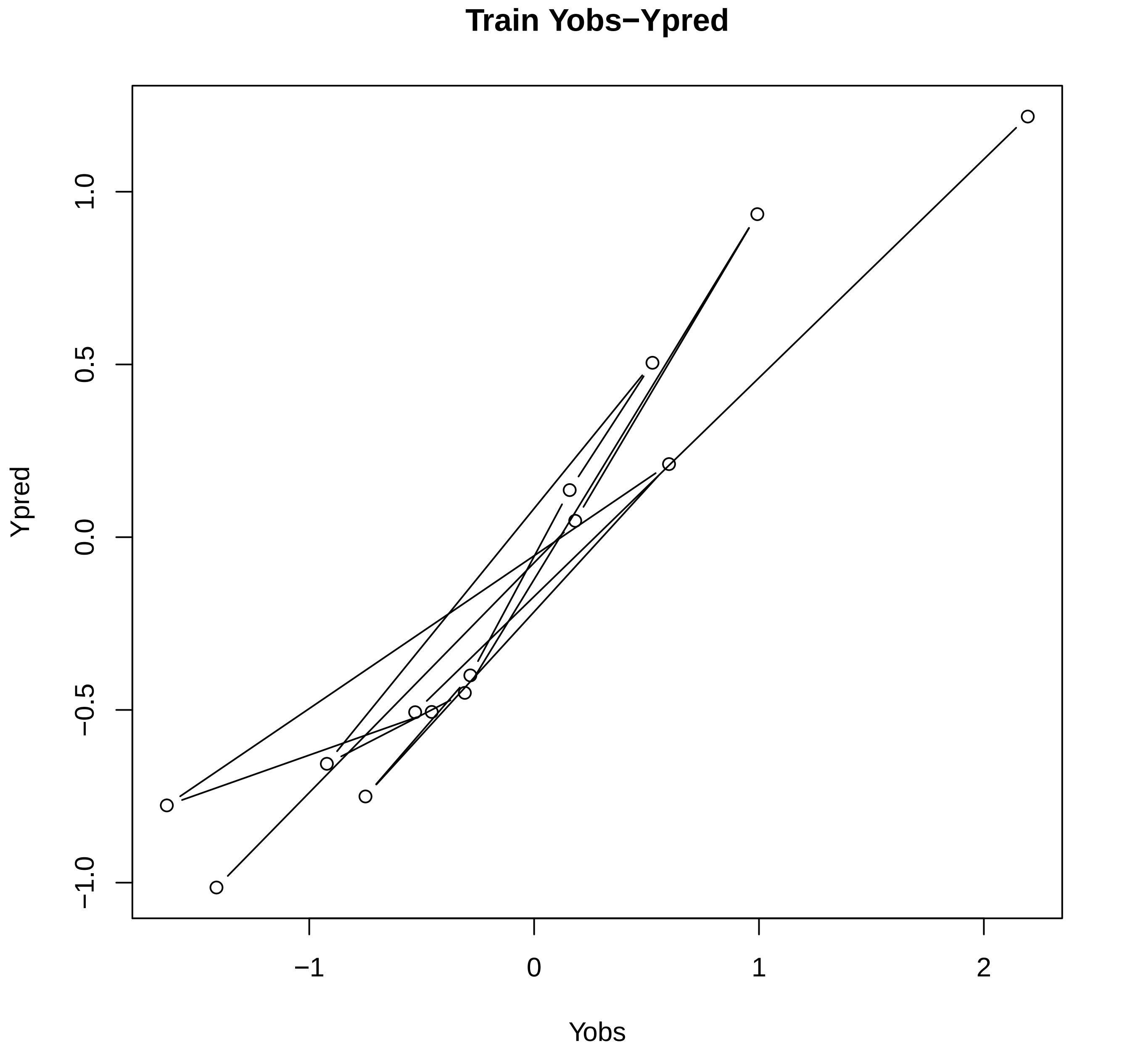

Train Yobs−Ypred
Ypred
Yobs
1.0
0.5
0.0
−0.5
−1.0
−1
0
1
2

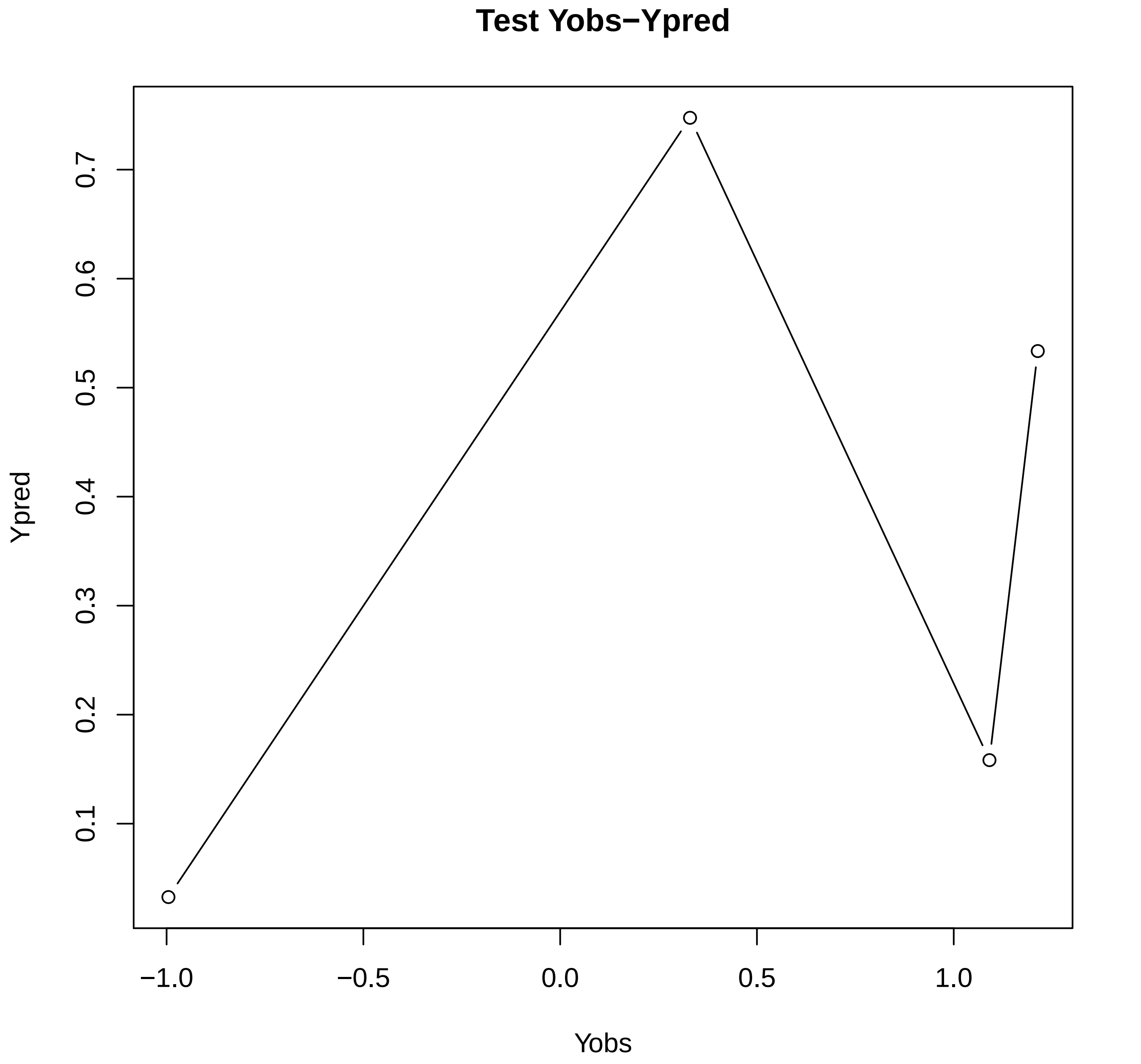
Test Yobs−Ypred
Ypred
0.1
0.2
0.3
0.4
0.5
0.6
0.7
Yobs
−1.0
−0.5
0.0
0.5
1.0

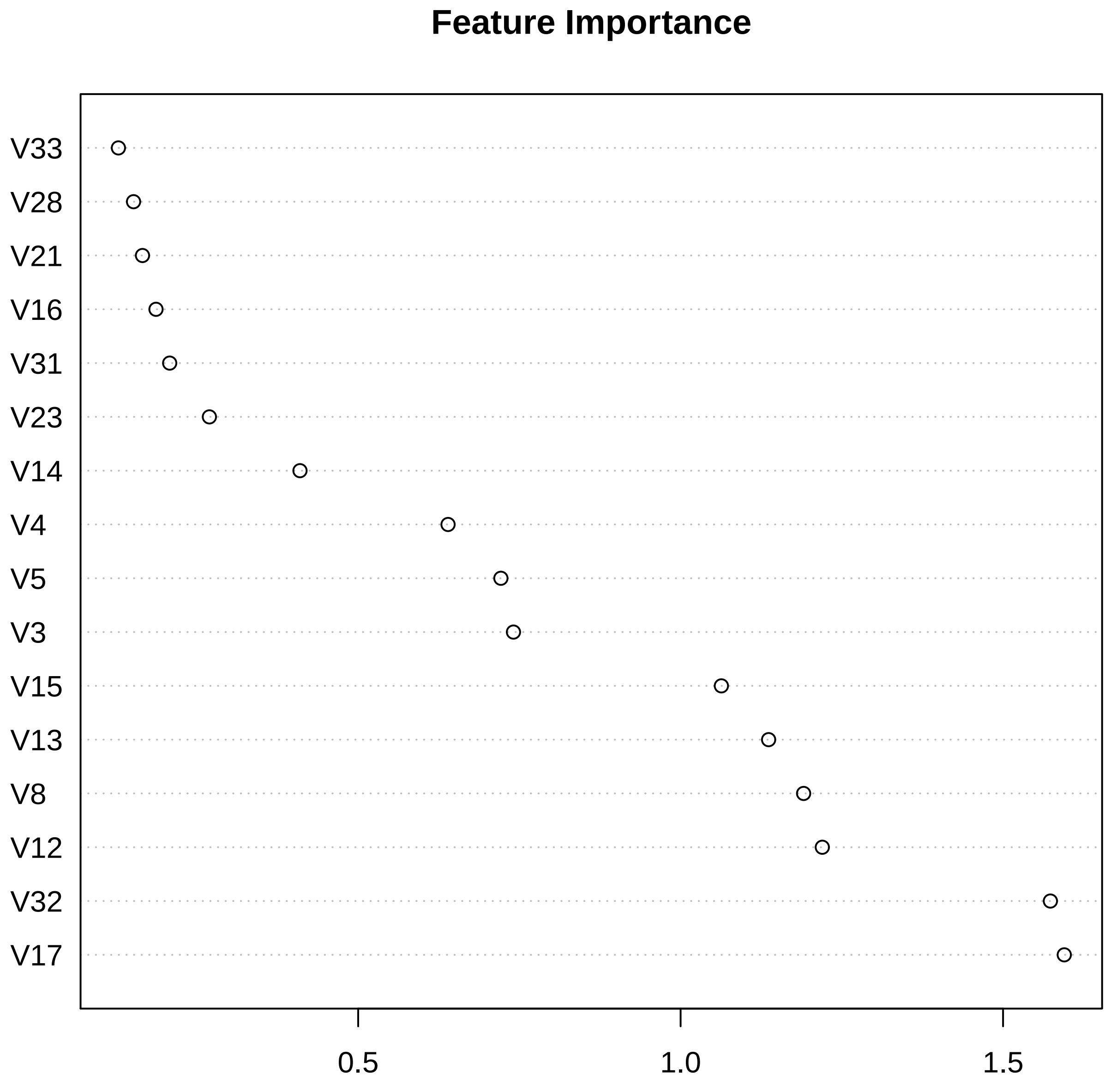
Feature Importance
V33
V28
V21
V16
V31
V23
V14
V4
V5
V3
V15
V13
V8
V12
V32
V17
0.5
1.0
1.5

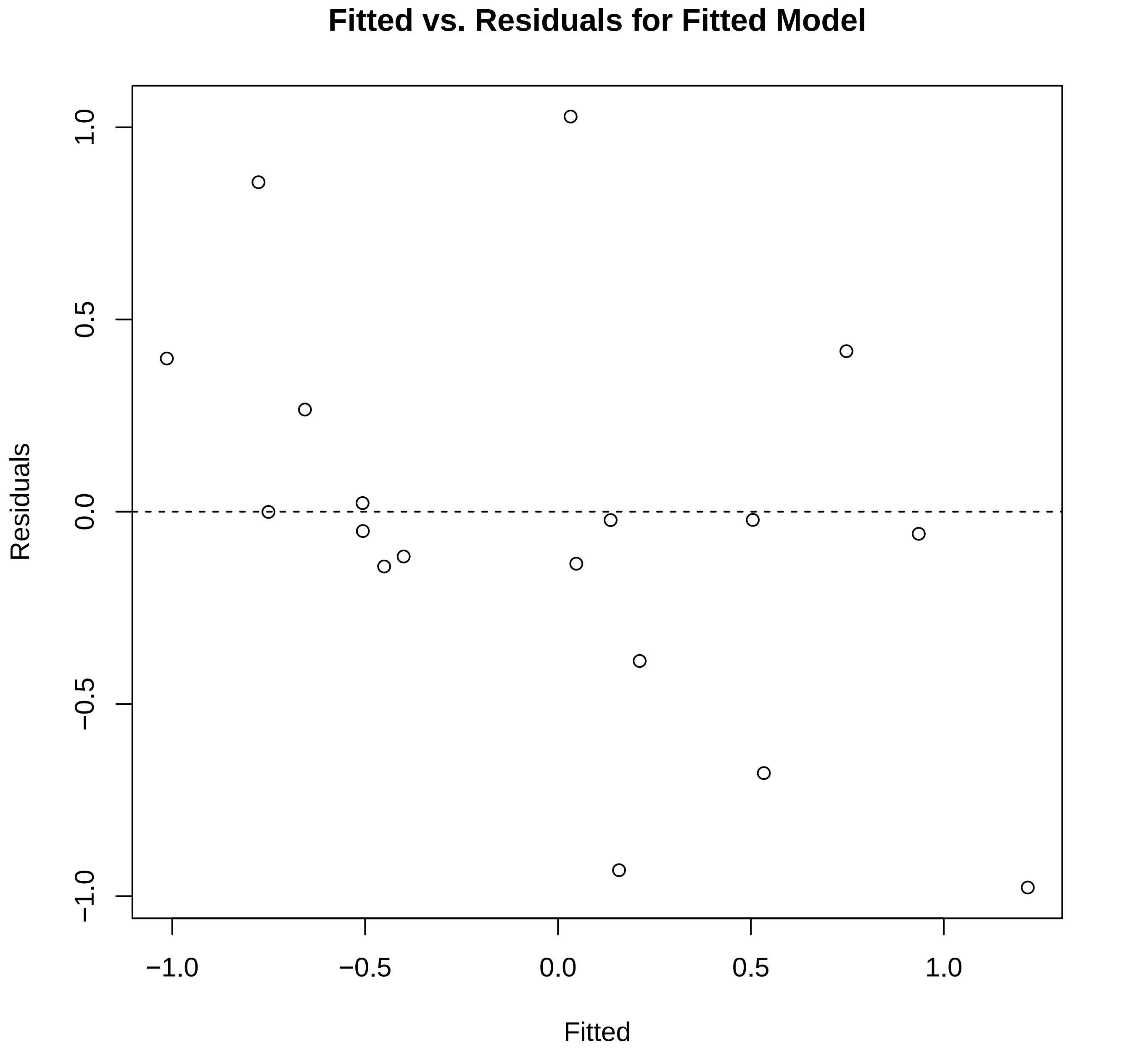
Fitted vs. Residuals for Fitted Model
Residuals
1.0
0.5
0.0
−0.5
−1.0
−1.0
−0.5
0.0
0.5
1.0
Fitted
